# 380. Implementation of Pooled Saliva Tests for Universal Screening of Congenital Cytomegalovirus (cCMV) Infection: Lessons Derived Over a 25-Months Period

**DOI:** 10.1093/ofid/ofae631.011

**Published:** 2025-01-29

**Authors:** Esther Oiknine-Djian, Smadar Eventov Friedman, Noa Ofek Shlomai, Moran Yassour, Dana Wolf

**Affiliations:** Hadassah Hebrew University Medical Center, Jerusalem, Yerushalayim, Israel; Hadassah Medical Center, Jerusalem, Israel, Jerusalem, Yerushalayim, Israel; Hadassah and Hebrew University Medical center, Jerusalem, Yerushalayim, Israel; The Hebrew University of Jerusalem, Jerusalem, Yerushalayim, Israel; Hadassah-Hebrew University Medical Center, Jerusalem, Yerushalayim, Israel

## Abstract

**Background:**

cCMV infection is the most common intrauterine infection, leading to infant neurodevelopmental disabilities and hearing loss, which can manifest at birth or develop later during childhood. Currently, targeted screening of cCMV by PCR testing of saliva samples is employed in many centers. However, targeted screening of high-risk neonates misses the majority of infants with cCMV who are asymptomatic at birth, yet are at risk for late-onset sequelae. Thus, universal newborn screening of cCMV has been increasingly advocated. In the absence of a high-throughput screening test that can identify all infected neonates, the development of an accurate and efficient testing strategy for universal cCMV screening has remained an ongoing challenge. We have recently reported on the development of a new saliva pooling setup for universal screening of cCMV (Nature Medicine 2024). Here we report on lessons derived over a 25-months period of its routine implementation.

**Methods:**

This prospective study took place in 2 hospitals of Jerusalem from April 2022 through April 2024. Saliva samples of all newborns with parental consent were tested for cCMV in 8-sample pools. Positive saliva results were confirmed by urine testing.

**Results:**

During the study period, 32,413 infants (94.4% of all live newborns) were screened for cCMV using the pooled approach. The pooling strategy proved highly efficient, with each PCR test covering an average of 6 newborns during the first 13 months of the study, thus reducing the number of required saliva tests by 83%. PCR loss of sensitivity was minor, in accordance with the theoretical prediction for an 8-sample pool. cCMV was identified in 123 newborns, with a birth prevalence of 3.8 per 1000. Over half (57.7%) of infants with cCMV identified by universal screening were asymptomatic at birth and would have been missed by targeted screening.

**Conclusion:**

The study demonstrates the wide feasibility and benefits of pooled-saliva cCMV testing as an accurate and cost-sparing approach, which could revolutionise the implementation of universal cCMV screening. Beyond the clinical implications toward early diagnosis, monitoring and treatment of cCMV, data derived from the implemented universal screening will serve to define the burden, risk factors, and long-term clinical outcomes of cCMV.

**Disclosures:**

**Dana Wolf, MD**, GSK: Advisor/Consultant|Merck: Grant/Research Support|Moderna: Grant/Research Support|MSD: Advisor/Consultant|Takeda: Advisor/Consultant

